# Neck exercises, physical and cognitive behavioural-graded activity as a treatment for adult whiplash patients with chronic neck pain: Design of a randomised controlled trial

**DOI:** 10.1186/1471-2474-12-274

**Published:** 2011-12-02

**Authors:** Inge Ris Hansen, Karen Søgaard, Robin Christensen, Bente Thomsen, Claus Manniche, Birgit Juul-Kristensen

**Affiliations:** 1 Research Unit for Musculoskeletal Function and Physiotherapy, Institute of Sports Science and Clinical Biomechanics, University of Southern Denmark, Odense M, Denmark; 2 Musculoskeletal Statistics Unit, The Parker Institute, Copenhagen University Hospital at Frederiksberg, Copenhagen F, Denmark; 3 Back Centre Southern Denmark, Hospital Lillebælt, Middelfart, Denmark

## Abstract

**Background:**

Many patients suffer from chronic neck pain following a whiplash injury. A combination of cognitive, behavioural therapy with physiotherapy interventions has been indicated to be effective in the management of patients with chronic whiplash-associated disorders. The objective is to present the design of a randomised controlled trial (RCT) aimed at evaluating the effectiveness of a combined individual physical and cognitive behavioural-graded activity program on self-reported general physical function, in addition to neck function, pain, disability and quality of life in patients with chronic neck pain following whiplash injury compared with a matched control group measured at baseline and 4 and 12 months after baseline.

**Methods/Design:**

The design is a two-centre, RCT-study with a parallel group design. Included are whiplash patients with chronic neck pain for more than 6 months, recruited from physiotherapy clinics and an out-patient hospital department in Denmark. Patients will be randomised to either a pain management (control) group or a combined pain management and training (intervention)group. The control group will receive four educational sessions on pain management, whereas the intervention group will receive the same educational sessions on pain management plus 8 individual training sessions for 4 months, including guidance in specific neck exercises and an aerobic training programme. Patients and physiotherapists are aware of the allocation and the treatment, while outcome assessors and data analysts are blinded. The primary outcome measures will be Medical Outcomes Study Short Form 36 (SF36), Physical Component Summary (PCS). Secondary outcomes will be Global Perceived Effect (-5 to +5), Neck Disability Index (0-50), Patient Specific Functioning Scale (0-10), numeric rating scale for pain bothersomeness (0-10), SF-36 Mental Component Summary (MCS), TAMPA scale of Kinesiophobia (17-68), Impact of Event Scale (0-45), EuroQol (0-1), craniocervical flexion test (22 mmHg - 30 mmHg), joint position error test and cervical range of movement. The SF36 scales are scored using norm-based methods with PCS and MCS having a mean score of 50 with a standard deviation of 10.

**Discussion:**

The perspectives of this study are discussed, in addition to the strengths and weaknesses.

**Trial registration:**

The study is registered in http://www.ClinicalTrials.gov identifier NCT01431261.

## Background

The Danish National Board of Health estimates that 5-6,000 subjects per year in Denmark are involved in a traffic accident evoking whiplash-induced neck pain. About 43% of those will still have physical impairment and symptoms 6 months after the accident [1]. For Swedish society, including Swedish insurance companies, the economic burden is approximately 320 million Euros [2], and this burden is likely to be comparable to that of Denmark. Most studies suggest that patients with Whiplash-Associated Disorders (WAD) report chronic neck symptoms one year after the injury [3]. The main problems in whiplash patients with chronic neck pain are cervical dysfunction and abnormal sensory processing, reduced neck mobility and stability, impaired cervicocephalic kinaesthetic sense, in addition to local and possibly generalised pain [4,5]. Cervical dysfunction is characterised by reduced function of the deep stabilising muscles of the neck.

Besides chronic neck pain, patients with WAD may suffer from physical inactivity as a consequence of prolonged pain [6,7]. This influences physical function and general health and can result in a poor quality of life. In addition, WAD patients may develop chronic pain followed by sensitisation of the nervous system [8,9], a lowering of the threshold for different sensory inputs (pressure, cold, warm, vibration and electrical impulses) [10]. This can be caused by an impaired central pain inhibition [11] - a cortical reorganisation [12]. Besides central sensitisation, the group with WAD may have poorer coping strategies and cognitive functions, compared with patients with chronic neck pain in general [13-15].

Studies have shown that physical training, including specific exercises targeting the deep postural muscles of the cervical spine, is effective in reducing neck pain [16-18] for patients with chronic neck pain, albeit there is a variability in the response to training with not every patient showing a major change. Physical behavioural-graded activity is a treatment approach with a focus on increasing general physical fitness, reducing fear of movement and increasing psychological function [19,20]. There is insufficient evidence for the long-term effect of treatment of physical and cognitive behavioural-graded activity, especially in chronic neck pain patients. Educational sessions, where the focus is on understanding complex chronic pain mechanisms and development of appropriate pain coping and/or cognitive behavioural strategies, have shown reduced general pain [6,21-26]. A review indicated that interventions with a combination of cognitive, behavioural therapy with physiotherapy including neck exercises is effective in the management of WAD patients with chronic neck pain [27], as also recommended by the Dutch clinical guidelines for WAD [28]. However, the conclusions regarding the guidelines are largely based on studies performed on patients with either acute or sub-acute WAD [29]. A more strict conclusion was drawn for WAD patients with chronic pain in the Bone and Joint Decade 2000-2010 Task Force, stating, that '*because of conflicting evidence and few high-quality studies, no firm conclusions could be drawn about the most effective non-invasive interventions for patients with chronic WAD*" [29,30]. The concept of combined treatment for WAD patients with chronic pain has been used in a former randomised controlled trial [31]. The results indicated that a combination of non-specific aerobic exercises and advice containing standardised pain education and reassurance and encouragement to resume light activity, produced better outcomes than advice alone for patients with WAD 3 months after the accident. The patients showed improvements in pain intensity, pain bothersomeness and functions in daily activities in the group receiving exercise and advice, compared with advice alone. However, the improvements were small and only apparent in the short term.

**Figure 1:**
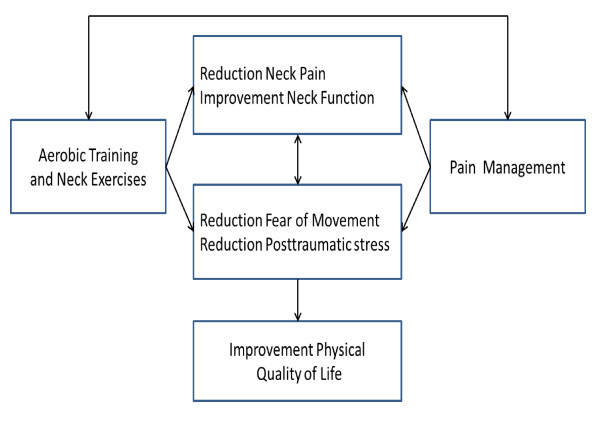
**Hypothesis of the intervention effect for patients with chronic neck pain after a whiplash accident**.

This project is formulated on the expectation that rehabilitation of WAD patients with chronic neck pain must target cervical dysfunctions, training of physical function and the understanding and management of chronic pain in a combined therapy approach. Each single intervention is based upon former studies that have shown effectiveness [6,18,20,32]. This study is the first to also include the long-term effect of the combined approach in patients with chronic neck pain after whiplash trauma. As illustrated in Figure 1, the conceptual model in this study is based upon the hypothesis that training (including both individually-guided specific neck exercises and graded aerobic training) and education in pain management (based on a cognitive behavioural approach) is better for increasing the patients' physical quality of life, compared with education in pain management alone. Increasing the physical quality of life includes increasing the general physical function and level of physical activity, decreasing fear of movement, reducing post-traumatic stress symptoms, reducing neck pain and increasing neck function. The effect is anticipated to be found immediately after the treatment (i.e. 4 months; short-term effect) as well as after one year (long-term effect).

Using a randomised controlled trial (RCT) design, the aim of this study is to evaluate the effectiveness of: graded physical training, including specific neck exercises and general aerobic training, combined with education in pain management (based on a cognitive behavioural approach) versus education in pain management (based on a cognitive behavioural approach), measured on physical quality of life', physical function, neck pain and neck functions, fear of movement, post-traumatic symptoms and mental quality of life, in patients with chronic neck pain after whiplash injury.

## Methods/Design

### Trial design

**Figure 2:**
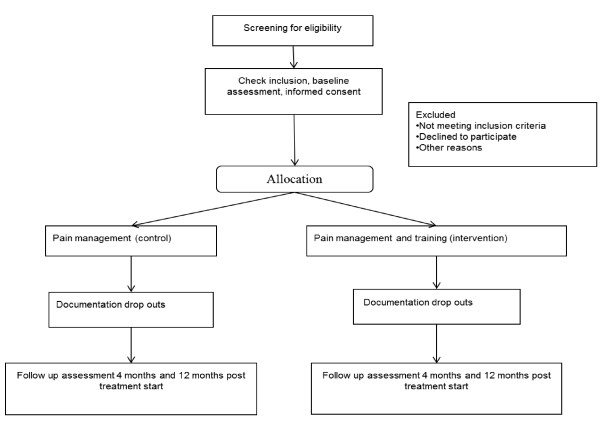
**Flowchart of the patients in the study**.

The study is conducted in Denmark as an RCT with a parallel group design. It will be a two-centre study, stratified by recruitment location. Patients will be randomised to either the Pain Management group (control) or the Pain Management and Training group (intervention). As illustrated in Figure 2, the study is designed to include a secondary data assessment 12 months after baseline; the primary outcome assessment will be performed immediately after the intervention program 4 months after baseline. The study utilises an allocation concealment process, ensuring that the group to which the patient is allocated is not known before the patient is entered into the study. The outcome assessors and data analysts will be kept blinded to the allocation to intervention or control group.

### Settings

The participants will be recruited from physiotherapy clinics in Denmark and from The Spine Centre of Southern Denmark, Hospital Lillebælt via an announcement at the clinics and the Hospital. Using physiotherapy clinics spread across Denmark, the patients will receive the intervention locally. The physiotherapy clinics in Denmark receive patients via referral from their general practitioners. The Spine Centre, a unit specialising in treating patients with musculoskeletal dysfunctions and only treating out-patients, receives patients referred from general practitioners and/or chiropractors.

### Study Population

Two hundred adults with a minimum age of 18 years, receiving physiotherapy treatment or having been referred for physiotherapy treatment will be recruited. For patients to be eligible, they must have: chronic neck pain for at least 6 months following a whiplash injury, reduced physical neck function (Neck Disability Index score, NDI, of a minimum of 10), pain primarily in the neck region, finished any medical /radiological examinations, the ability to read and understand Danish and the ability to participate in the exercise program. The exclusion criteria include: neuropathies/ radiculopathies (clinically tested by: positive Spurling, cervical traction and plexus brachialis tests) [33], neurological deficits (tested as in normal clinical practice through a process of examining for unknown pathology), engagement in experimental medical treatment, being in an unstable social and/or working situation, pregnancy, known fractures, depression according to the Beck Depression Index (score > 29) [18,34,35], or other known coexisting medical conditions which could severely restrict participation in the exercise program. The participants will be asked not to seek other physiotherapy or cognitive treatment during the study period.

### Intervention

#### Control

*The Pain Management (control) group *will receive education in pain management strategies. There will be 4 sessions of 11/2 hours, covering topics regarding pain mechanisms, acceptance of pain, coping strategies, and goal-setting, based upon pain management and cognitive therapy concepts [21,26,36].

#### Intervention

*The Pain Management plus Training (intervention) group *will receive the same education in pain management as those in the control group plus 8 treatment sessions (instruction in neck exercises and aerobic training) with the same period of 4 months length. If the treating physiotherapist estimates additional treatments are needed, the treatment can be extended with 2 more sessions. ***Neck training*: **The treatment of neck-specific exercises will be progressed through different phases, which are defined by set levels of neck function. At the first treatment session, patients are tested for cervical neuromuscular function to identify the specific level at which to start neck training. A specific individually tailored exercise program will be used to target the neck flexor and extensor muscles. The ability to activate the deep cervical neck flexor muscles of the upper cervical region to increase their strength, endurance and stability function is trained progressively via the craniocervical training method using a biopressure feedback transducer [18,37]. Exercises for neck-eye coordination, neck joint positioning, balance and endurance training of the neck muscles will be included as well, since it has been shown to reduce pain and improve sensorimotor control in patients with insidious neck pain [17,38]. ***Aerobic training: ***The large trunk and leg muscles will be trained with a gradually increasing physical training program. Patients will be allowed to select activities such as walking, cycling, stick walking, swimming, and jogging. The baseline for training duration is set by exercising 3 times at a comfortable level, that does not exacerbate pain and aims at a rated perceived exertion (RPE) level of between 11 and 14 on a Borg scale [39]. The initial duration of training is set 20% below the average time of the three trials. Training sessions are carried out every second day with a prerequisite that pain is not worsened, and that RPE is between 9 and 14. A training diary is used. If patients do not experience a relapse, and report an average RPE value of 14 or less, the exercise duration for the following period (1 or 2 weeks) is increased by 2-5 minutes, up to a maximum of 30 minutes. If the RPE level is 15 or higher, the exercise duration will be reduced to an average RPE score of 11 to 14 every fortnight [20,40]. By using these pacing principles, the training will be graded individually by the patient, with a focus on perceived exertion - with the aim of increasing the patient' s general physical activity level and fitness.

Patients' compliance will be administered by registration of their participation in the control and intervention group. The patients in the control group will be considered to have completed the pain management if they have attended 3 out of 4 sessions. The patiesnts in the intervention group will be considered to have completed if the patient has attended a minimum of 3 out of 4 pain management sessions and a minimum of 5 out of 8 trainings sessions. Each patient's home training with neck exercises and aerobic training will be registered by him/her in a logbook. Compliance with 75% of the planned home training will be considered as having completed the intervention.

### Physiotherapists

The participating physiotherapists will be recruited via an announcement in the Danish Physiotherapy Journal. The inclusion criteria consist of: being a qualified physiotherapist, working at a clinic and having at least two years of working experience as a physiotherapist, having attended a course in the described intervention and passed the related exam.

### Outcome measures

At baseline the participants' information on age, gender, height and weight, type of accident, medication, development of symptoms over the last two months (status quo, improving, worsening), expectation of treatment, employment and educational status will be registered. As a primary outcome measure, Medical Outcomes Study Short Form 36 (SF36) - Physical Component Summary (PCS) will be used [41,42]. The PCS scales are scored using norm-based methods [43,44] with a mean score of 50 with a standard deviation of 10. The primary outcome with respect to having an effect, will be calculated as a change from baseline [45]. Secondary outcomes contain data on both clinical tests and patient-reported outcomes. Table 1 presents clinical tests for measuring the intervention effect on neuromuscular control of the cervical muscles, cervical function and mechanical allodynia. Table 2 presents the patient-related outcomes from questionnaires used to test for perceived effect of the treatment, neck pain and function, pain bothersomeness, fear of movement, post-traumatic stress and quality of life and potential treatment modifiers.

**Table 1:** Clinical outcomes used for measurement of treatment effect on muscle strategy, function and treatment modifiers

Test description	Purpose
The craniocervical flexion test [50], measured with biopressure feedback transducer (range22-30 mmHg)	To test changed neuromuscular control of the neck

Neck joint position error test [51,52], measured with a laser pointing at a target in cm	To test change in neck positioning sense

Gaze stability test [17,53] measured positive when provoking symptoms as dizziness, nausea or changes in vision or an inability to maintain focus	To test change in head and eye coordination

Cervical range of movement [54], measured with an inclinometer in degrees	To test change of neck mobility

Mechanical allodynia [55-57] measured with Pressure Pain Threshold (PPT) transducer in selected standardised tender points in different body regions	To test for potential treatment modifiers

**Table 2:** Patient reported outcomes used for measured of treatment effect on pain and function

Outcome name	Purpose
Self-rated measure Global Perceived Effect (GPE) of global impression of recovery, with the question: "Compared to when this treatment first started, how would you describe your neck this last week?" (range 0-11) where minus five represents vastly worse, 0 represents unchanged, and five represents completely recovered (Kamper et al., 2010, Stewart et al., 2007a, Geisser et al., 2010) (range 0-11)	To measure perceived change

Neck Disability Index (NDI) [49], with higher scores representing greater perceived disability [58-60] (range 0-50)	To test for change in neck pain and neck disability, related to daily activities

Pain Specific Functioning Scale, comprising three patient-rated important activities, rated from the level of difficulty with lower scores representing better function (range 0-10 scale)	To test for change in self- reported function

Numerical rating scale, with higher scores representing greater pain bothersomeness, (Stewart et al., 2007a, Young et al., 2010) (range 0-10)	To test for change in pain bothersomeness

TAMPA Scale of Kinesiophobia (fear of re-injury due to movement), a psychological distress questionnaire, with a score above 37 indicating a high degree of kinesiophobia (Cleland et al., 2008, Nederhand et al., 2004, Nederhand et al., 2006) (range 17-68),	To test for change in fear of movement

The Impact of Events Scale, [61,62] (range 0-75)	To test for change in post-traumatic stress symptoms

The Euroqol-5D capturing the patient's perceived state of health, with predefined end-points, high value is good health and low value is bad health [63,64] (range 0-100)	To test for change in quality of life

Patients will be tested at baseline, 4 and 12 months after baseline, except for GPE, which will only be measured 4 and 12 months after baseline.

### Power and sample size estimation

The power and sample size calculation is based on the primary outcome, being SF36-PCS 4 months after baseline. For a two-sample pooled t-test of a normal mean difference with a two-sided significance level of 0.05, assuming a common SD of 10, a sample size of 86 per group is required to obtain a power of at least 90% to detect a group mean difference of 5 PCS points [45]; the actual power is 90.3%, and the fractional sample size that achieves a power of exactly 90% is 85.03 per group. In order to adjust for an estimated 15% withdrawal during the study period of 4 months, we will include 100 patients in each group. For sensitivity, three scenarios were applied: firstly, anticipating that all 2 × 100 patients complete the trial, we will have sufficient power (> 80%) to detect a group mean difference as low as 4 PCS points; secondly, we will be able to detect a statistically significant group mean difference of 5 PCS points with sufficient power (> 80%) even with a pooled SD of 12 PCS points. Thirdly and finally, if we aim for a group mean difference of 5 PCS points, with a pooled SD of 10, we will have sufficient power (> 80%) with only 64 patients in each group. However, for logistical reasons, new patients will no longer be included in the study 24 months after the first patient has been included.

### Randomisation, allocation and blinding procedures

After the baseline assessment, the participants are randomly assigned to either the control group or the intervention group. The randomisation sequence is created using SAS (SAS 9.2 TS level 1 M0) statistical software and is stratified by centre with a 1:1 allocation using random block sizes of 2, 4, and 6. The allocation sequence will be concealed from the researcher enrolling and assessing participants in sequentially numbered, opaque, sealed and stapled envelopes. Aluminium foil inside the envelope will be used to render the envelope impermeable to intense light. After revealing the content of the envelope, both patients and physiotherapists are aware of the allocation and the corresponding treatment. Outcome assessors and data analysts are however kept blinded. Prior to the outcome assessments, the patients will be asked by the research assistant not to mention the treatment to which they have been allocated.

### Statistical analysis

All the primary data analyses will be carried out according to a pre-established analysis plan; all analyses will be done applying SAS software (v. 9.2 Service Pack 4; SAS Institute Inc., Cary, NC, USA). All descriptive statistics and tests are reported in accordance with the recommendations of the 'Enhancing the QUAlity and Transparency Of health Research' (EQUATOR) network; i.e., various forms of the CONSORT statement [46]. Data will be analysed using a two-factor Analysis of Covariance (ANCOVA), with a factor for Group and a factor for Gender, using the baseline value as covariate to reduce the random variation, and increase the statistical power. Unless stated otherwise, results will be expressed as the difference between the group means with 95% confidence intervals (CIs) and associated p-values, based on a General Linear Model (GLM) procedure. All the analyses will be performed using the Statistical Package for Social Sciences (version 19.0.0, IBM, USA) as well as the SAS system (v. 9.2; SAS Institute Inc., Cary, NC, USA). A two-way analysis of variance (ANOVA) with repeated measures (Mixed model) will be performed to test the difference over time between the intervention and the control groups; interaction: Group × Time. An alpha-level of 0.05 will be considered as being statistically significant (p < 0.05, two- sided). The data analysts will be blinded to the allocated interventions for primary analyses.

The baseline scores for the primary and secondary outcomes will be used to compare the control and intervention groups. The statistical analyses will be performed on the basis of the intention-to-treat principle, i.e. patients will be analysed in the treatment group to which they were randomly allocated. In the primary analyses, missing data will be replaced with the feasible and transparent 'Baseline Observation Carried Forward' (BOCF) technique, and for sensitivity also a multiple imputation technique will apply.

Secondarily, to relate the results to compliance, a 'per protocol' analysis will be used as well. The 'per protocol' population he patients who have 'completed' the intervention to which they were allocated, according to the principles described in the intervention section above.

### Ethical considerations

The Regional Scientific Ethical Committee of Southern Denmark approved the study (S-20100069). The study conformed to The Declaration of Helsinki 2008 [47] by fulfilling all general ethical recommendations.

All subjects will receive information about the purpose and content of the project and give their oral and written consent to participate, with the possibility to drop out of the project at any time.

## Discussion

This study will contribute to a better understanding of treating patients with chronic neck pain following a whiplash accident. The knowledge from this study can be implemented into clinical practice, as the study is based on a multimodal approach, mirroring the approach, which in spite of the current lack of evidence, is often used in a clinical physiotherapy setting. The study may also be included in systematic reviews thereby contributing to updating the knowledge about this population and to enhancing evidence-based treatment.

Publishing the design of a study before the study is performed and the results obtained has several advantages. It allows the design to be finalised without its being influenced by the outcomes. This can assist in preventing bias as deviations from the original design can be identified. Other research projects will have the opportunity to follow a similar approach with respect to population, interventions, controls and outcome measurements. The challenges of this study are related to standardising the interventions, treating a non-homogeneous population, defining and standardising relevant outcome measures on a population with long-lasting symptoms and having a population from two different clinical settings. Standardisation of the interventions is obtained by teaching the involved physiotherapists in an instructional course. Population homogeneity will be handled by strict inclusion and exclusion criteria and by monitoring the baseline characteristics of the patients, and differences between groups based on other influences than the intervention/control will be possible to analyse statistically. This research design is composed as an 'add-on' design: both groups receive pain education; the intervention group receives additional physical training, including specific neck exercises and general training. Today there is insufficient evidence for the effect of treatment for patients with chronic neck pain following a whiplash accident. All participating patients will be referred for a treatment (control or intervention), as we consider it unethical not to offer some form of treatment, i.e. randomising the control group to a waiting list. The add-on design is chosen as a pragmatic workable solution in such a situation [48].

For whiplash patients with chronic pain, the most responsive disability measures (for the individual patient, not for the group as a whole) are considered to be the Patient Specific Functional Scale and the numerical rating scale of pain bothersomeness [49]. By using these and NDI (the most often used neck disability measure) as secondary outcome measures, it is anticipated that patient-relevant changes in pain and disability can be evaluated. The population will be recruited from and treated at two different clinical settings: the out-patient clinic of The Spine Centre, Hospital Lillebælt and several private physiotherapy clinics. To avoid any influence of the different settings on the outcome measures, the population will be block randomised related to the settings, securing equal distribution of participants from each setting to the two intervention groups.

## Competing interests

The authors declare that they have no competing interests.

## Authors' contributions

IRH drafted the manuscript. IRH, BJK and KS participated in the design of the study. All contributed to the design. RC, IRH; BJK and KS participated in the power and sample size calculation and in describing the statistical analysis as well as the allocation and randomization procedure. All authors read and approved the final manuscript. Suzanne Capell provided writing assistance and linguistic corrections.

## Pre-publication history

The pre-publication history for this paper can be accessed here:

http://www.biomedcentral.com/1471-2474/12/274/prepub
